# Consumer Wearable Deployments in Actigraphy Research: Evaluation of an Observational Study

**DOI:** 10.2196/12190

**Published:** 2019-06-24

**Authors:** Ciara Duignan, Patrick Slevin, Niladri Sett, Brian Caulfield

**Affiliations:** 1 Insight Centre for Data Analytics University College Dublin Dublin Ireland; 2 School of Public Health, Physiotherapy and Sports Science University College Dublin Dublin Ireland

**Keywords:** wearable electronic device, digital divide, activity trackers, technology, wearable challenges

## Abstract

**Background:**

Consumer wearables can provide a practical and accessible method of data collection in actigraphy research. However, as this area continues to grow, it is becoming increasingly important for researchers to be aware of the many challenges facing the capture of quality data using consumer wearables.

**Objective:**

This study aimed to (1) present the challenges encountered by a research team in actigraphy data collection using a consumer wearable and (2) present considerations for researchers to apply in the pursuit of robust data using this approach.

**Methods:**

The Nokia Go was deployed to 33 elite Gaelic footballers from a single team for a planned period of 14 weeks. A bring-your-own-device model was employed for this study where the Health Mate app was downloaded on participants’ personal mobile phones and connected to the Nokia Go via Bluetooth. Retrospective evaluation of the researcher and participant experience was conducted through transactional data such as study logs and email correspondence. The participant experience of the data collection process was further explored through the design of a 34-question survey utilizing aspects of the Technology Acceptance Model.

**Results:**

Researcher challenges included device disconnection, logistics and monitoring, and rectifying of technical issues. Participant challenges included device syncing, loss of the device, and wear issues, particularly during contact sport. Following disconnection issues, the data collection period was defined as 87 days for which there were 18 remaining participants. Average wear time was 79 out of 87 days (90%) and 20.8 hours per day. The participant survey found mainly positive results regarding device comfort, perceived ease of use, and perceived usefulness.

**Conclusions:**

Although this study did not encounter some of the common published barriers to wearable data collection, our experience was impacted by technical issues such as disconnection and syncing challenges, practical considerations such as loss of the device, issues with personal mobile phones in the bring-your-own-device model, and the logistics and resources required to ensure a smooth data collection with an active cohort. Recommendations for achieving high-quality data are made for readers to consider in the deployment of consumer wearables in research.

## Introduction

The advancement of wearable technology has brought with it the promise of expanding the capabilities of health care. Wearable sensors have the potential to augment and transform the diagnosis and ongoing management of both physical and physiological conditions. Due to this potential, wearables have been used increasingly for research in many areas of health and performance science. Recently, we note the progression of this work to incorporate activity tracking and its association with behavioral support and biological markers, respectively [1-3]. Although fitness trackers have been shown to increase physical activity levels, current evidence does not support their use in improving health outcomes beyond clinical interventions [4,5]. There has been a rapid growth of registered studies using consumer wearables, thus the body of published work in this area is expected to continue to mature [6].

Consumer wearables are a familiar, accessible, and cost-effective solution for remote actigraphy measurement, making them an attractive option for research studies [7,8]. However, ensuring the capture of high-quality data with consumer wearables presents a growing challenge. Frequently cited challenges include usability problems, unreliable technology, activity detection, limited device battery power, and user adherence [9,10]. Adherence is a crucial barrier to data capture and can be influenced by factors such as device convenience and comfort, and its charging, complexity, and interaction requirements [10]. User interest and motivation can be central to adherence and can be further determined by aspects such as the perceived usefulness of the device [11].

Knowledge regarding the challenges of data capture using wearables represents a pressing need, especially as research and industry deployments continue to grow in rapidly advancing areas such as digital health and connected health. If fresh understandings are created regarding how to implement, deploy, organize, and execute a wearable-led study, there is an opportunity to enhance deployments and avoid the creation of an unsatisfactory experience and unsuccessful outcomes for researcher and participant.

The aim of this study was to present and discuss the challenges experienced in a longitudinal data collection using a consumer wearable device with a healthy, active cohort where initial recruitment had been established. Second, the study will aim to identify how we might mitigate these issues, with the desired outcome being recommendations to promote high-quality data collection using consumer wearables.

This study was not originally designed to investigate the challenges of deploying a consumer wearable, rather, we set out to conduct a larger observational study where actigraphy was one of the measures included in our data collection. The content presented here has emerged from the experience of conducting this study, where we outline our observations and evaluation of the data capture process.

## Methods

### Part 1: Original Study

#### Study Design

The original study conducted was a longitudinal observation of activity and wellness measures in elite Gaelic footballers over a single season. Objective activity and sleep measures were obtained via a consumer wearable device, and self-reported wellness measures were recorded via a commercial mobile application daily. A total of 34 male elite Gaelic footballers (age: mean 23.4, SD 2.8 years) from a single team were recruited as a purposeful sample for this study and were eligible to take part if they were older than 18 years, part of the senior football team, and had the ability to provide informed consent. There were no exclusion criteria. Ethical approval for this study was granted by University College Dublin Human Research Ethics Committee. Participants were required to provide informed written consent before participation and were advised of their right to withdraw from the study at any time.

#### Device

Participants were an active group of sportspeople; a diverse cohort including students and working professionals in both sedentary and active jobs. Due to the duration of the data collection and the nature of the cohort, the device requirements were intended to be passive and involve as little disruption as possible for the participants. The user requirements for the device were defined as (1) comfortable and unobtrusive; (2) durable and water resistant, with a secure clasp; (3) long battery life (noncharging); and (4) clock function. The authors searched for suitably neat wrist-worn wearables and researched the features as required (Table 1).

**Table 1:** Device comparison.

Device name	Replaceable battery	Water resistant	Clock function
Fitbit Flex	No	No	Yes
Fitbit Flex 2	No	Yes	No
Polar Loop	No	Yes	Yes
Jawbone UP	No	No	No
Jawbone UP 2	No	No	No
Misfit Ray	Yes	Yes	No
Misfit Shine	Yes	Yes	Yes
Archon Touch	No	No	Yes
Striiv	No	No	Yes
Garmin Vivofit 2	Yes	Yes	Yes
Nokia Go	Yes	Yes	Yes

**Figure 1:**
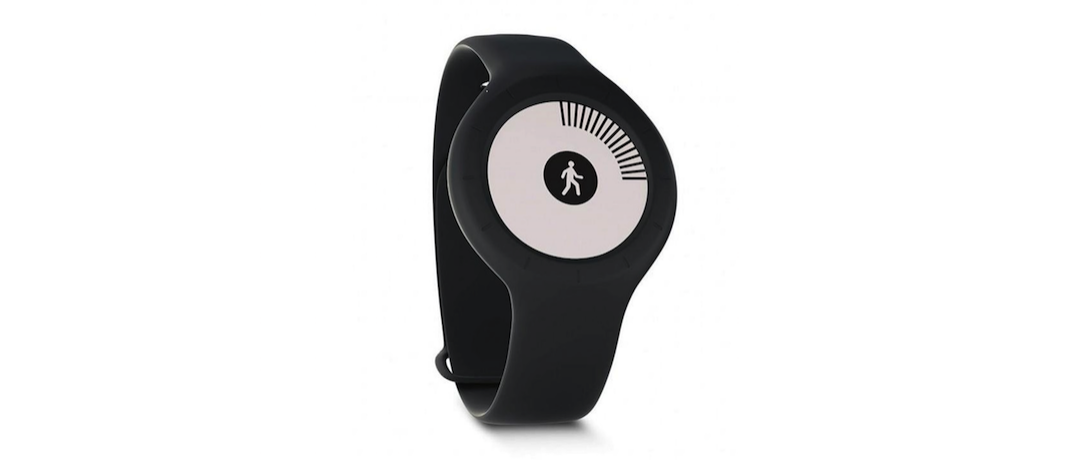
The Nokia Go wearable activity and sleep tracker.

The study device was not limited to any consumer or medical grade device; however, it was necessary to access raw intraday data from the device, requiring an open application programming interface (API). Access to the Garmin API carries a substantial fee; therefore, the Garmin Vivofit 2 was not considered for selection. The authors trialed the 2 devices that fit the defined criteria (Misfit Shine and Nokia Go) before choosing to provide participants with the Nokia Go because of its perceived durability and comfort during exercise. The Nokia Go (Nokia Technologies, Finland) is a wrist-worn activity and sleep tracker. Form factor design is a watch shape, with an E Ink display of your activity progression, which can also display an analog clock when pressed. The body of the device sits tightly into the strap, and the clasp is a thread-through with a button press. The watch is water resistant up to 50 m with automatic activity and sleep recognition and a replaceable battery lasting up to 8 months (Figure 1).

#### Data Collection

The study design required participants to wear the device for 24 hours a day over 2 specified periods: Preseason, which was 6 weeks spread across November and December and in-season, which was 8 weeks in February and March. The Health Mate app (Nokia Technologies, Finland) was downloaded and installed on each participant’s personal mobile phone. A confidential research account email address and password was set up for each participant, and their device was connected to the Health Mate app on their personal mobile phone via Bluetooth. Outcomes of interest in this study were step count and sleep duration; therefore, no additional app settings were applied (such as body mass index). Deployment was conducted during the preseason phase of competition by the lead researcher. Access to the account data was set up via Nokia’s API.

Daily individual step count was recorded through the API to monitor device wearing and syncing by participants. The Nokia Go device purported to sync with the Health Mate app automatically, provided Bluetooth was enabled on the mobile device. Participants were advised to sync the watch daily if they did not have Bluetooth always enabled on their mobile phone. If a participant had no data recorded on the Health Mate app for that day, the lead researcher would contact them with reminders to wear and sync the device. The device was to be worn at all times with the exception of during competitive Gaelic games, where jewelry wear is not permitted because of health and safety reasons. Participants were required to have data collected on at least 75% (65) of the study days to be included in the original study analysis.

### Part 2: Retrospective Study Evaluation

During the data collection for the original study, considerable challenges were encountered by the study team. We subsequently decided to review and interrogate these issues through retrospective researcher and participant analysis, which were not part of the original study design. The results have, therefore, been structured into 4 sections: sections 1 and 2 detail the researcher and participant challenges, which were collated from transactional data such as research team correspondence, study logs, and messages from participants, whereas the third section outlines the actual data collected as part of the original study design, and the fourth was derived from the participant survey, which was designed as follows: A 34-question survey was designed to further understand the user experience of study participation and gain insight into data collection challenges from the participant’s perspective. Survey design leveraged the Technology Acceptance Model [12], that is, perceived usefulness, perceived ease of use, attitude toward use, behavioral intention to use, and actual use, in addition to some practical questions. In 12 questions, a positive statement was made, and participants were asked to rate their agreement on a 5-point Likert scale. A total of 9 questions were multiple choice, 3 questions were open ended, and a further 10 questions were follow-up questions. The anonymous survey was deployed to participants online via Google Forms in the month following conclusion of the study (see Multimedia Appendix 1).

## Results

### Researcher Challenges

#### Disconnection and Product Support

The devices connected to each of the participants’ phones without issue, with the exception of 1 Huawei PRA-LX1, which was listed on the Nokia website as incompatible. Therefore, 33 participants were included in the device deployment in November 2017. In a 9-day period, 20 participants experienced a disconnection. Disconnection happened between 3 to 12 days after deployment and had no pattern to phone type. This was generally reported by participants as “my watch won’t sync” or “sleep data isn’t working.” On investigation, it appeared that the devices had disassociated from the user account. On the devices’ page of the Health Mate app, where the connected device normally appeared, there was no device connected. The Nokia Go appeared to be recording data as shown on the watch display, but this data could not be collected as we were unable to sync it to a phone without performing a factory reset.

Where participants reported that their “sleep data isn’t working,” it transpired that the Nokia Go had disconnected from the Health Mate app, but the app had switched to collecting step data from the phone. This meant that many participants did not recognize there was an issue and subsequently, did not report the problem immediately. It was also difficult for the researchers to identify these anomalies; as to us, the participant may just have not been wearing the device at night.

At one point during data capture, the Nokia API requirements for accessing summary activity data changed. There had been no prior announcement from Nokia regarding this change, and the cause of the problem was discovered by the authors through developer chat rooms. In addition, it took multiple requests from the research team to Nokia product support to gain authorization for accessing intraday activity through the API.

#### Logistics

The research plan was to deploy the devices for 6 weeks in preseason (November-December 2017) as a trial period whereby we would assess feasibility and iron out potential issues and thus, redeploying the devices for an 8-week data collection during in-season (February-March 2018). After the initial disconnection issue, we reset and resynced the devices with the hope that this would be a once off issue. This took up to 3 weeks by the time the issue was identified and rectified in each case. One disassociated device would not reconnect; again, this was a Huawei phone but not a model listed as incompatible on the Nokia website.

When issues arose in the first 9 days of the study, we were very aware of the burden on participants in reporting and rectifying these issues. Potentially, the initial novelty of participation was dissipated during that time, and the logistics of collecting and redeploying the devices seemed like a process that would have been burdening on participants and challenging to maintain compliance. At this point we made the decision to continue the data collection uninterrupted from initial deployment (November 2017) to the end of the study (March 2018).

When researchers contacted participants to remind them to wear the device, it became apparent that although participants were wearing the device, they had not manually synced it or did not have Bluetooth enabled on their phone for automatic syncing. Participants were already receiving a daily reminder for another action as part of the wider study participation, and we became cognizant of the burden and potential annoyance of this contact, particularly as the data collection period had been extended. We made the decision to reduce syncing reminders to once a week, but often, this meant we could lose data if in fact the participant was not wearing the device.

The lead researcher (CD) worked clinically with the participant group 3 times per week on average, and when problems or technical issues arose, she planned to address these during her clinical visits. This was often impeded, however, by simple barriers such as the participant forgetting their phone, their phone battery being depleted, their phone not having internet access because of data restrictions, and frequently, they would forget to bring the watch, having taken it off when it appeared to or did stop working. Many participants also lost or had issues with their mobile phone over the course of the study, which resulted in lost data. Some participants changed their mobile phone, which required the action of another setup process, and again, may have resulted in lost data if they did not inform the lead researcher before changing.

### Participant Challenges

#### Syncing

As many of the participants did not normally leave Bluetooth enabled on their mobile phone, they were required to manually sync the device daily. This method was suboptimal in that it required an action from the participant, which they would often forget to do. Participants reported that syncing could be very slow, and if the phone screen timed out, it would lead them to believe the sync had not worked; a belief which was reinforced for many by the initial disassociation. In addition, for those who did have their Bluetooth enabled, Nokia support informed us that “If you occasionally turn off Bluetooth on your device, it is possible that the Nokia Go will stop syncing even after Bluetooth is turned back on. If you encounter this issue, you will need to Force Stop the Health Mate app, turn on Bluetooth, and then launch the Health Mate app again,” meaning that automatic syncing did not occur for the entire study. When the device stopped syncing automatically for those participants, their study participation routine changed from a passive process to one that required daily action.

#### Wearing

Anecdotally, the device appeared to be acceptable for most participants. There was 1 dropout from the study. This participant was a teacher and cited his decision in the comment section of the survey as occupational impracticality: “As a practical teacher one of the key things in each lesson is having the room tidied before the bell. The watch was not functional in this sense. In particular, the clock would twist in the socket and I would be reading the time wrong by a few minutes.”

Loss of the device was a common issue with 7 permanent losses reported over the study duration, 3 of which were during the Christmas period. Feedback from the participants on how this happened is presented in the survey results section below. A total of 3 participants opted not to wear the device during sport, citing glove wear and tackling as a reason. Early in the study, it appeared that the devices were being mislaid during sport because of the watch falling off during a tackle or similar contact. Some of them were retrieved and handed to a staff member, and others were not found. At this point it was decided that participants should no longer wear the device during sport. Although this was going to disrupt data collection, it appeared to be more desirable than losing further devices. As expected, many participants would either forget to remove the device for sport or forget to put it back on afterwards. The disruption this caused to data collection also meant that overall step counts were no longer reliable, as some could reflect training activity, and some may not. Furthermore, regulating wear during training was not feasible under the study conditions.

There was 1 reported instance of 2 participants confusing each other’s device for their own. Although devices were marked with a study code by both waterproof stickers and permanent marker, neither method stood the test of time with this cohort.

### Actual Data Collected

After rectifying the initial dissociation issues, the data collection period was defined as a total of 87 days from January to March 2018. A total of 9 participants were lost from the study because of cuts to the team panel, injury, and personal reasons, which were beyond our control. As previously mentioned, 1 device would not reconnect to a participants’ mobile phone after the initial dissociation, and another had technical difficulties with syncing the device. A total of 4 participants lost their device during this data collection period, which totals to 6 participants who were removed from the cohort as they did not reach the 75% threshold of data collection.

Therefore, 18 of the remaining 24 participants were included in the analysis of the full data collection period. Nonwear time was calculated by considering 90 min of inactivity as the cut-off for nonwear [13]. The average overall wear was 79 out of 87 days (90%). On those days when the device was worn, the average wear time was 20.8 hours/day. Average wear time was calculated using a weighted average (by wear days) of the individual players.

### Participant Feedback (Survey)

The 34-question feedback survey was deployed online via Google forms to participants in the month following completion of the study with a response rate of 91% (30/33). Results from the multiple-choice questions are presented in Multimedia Appendix 2.

#### Practical Considerations: Follow-Up Questions

Of those who did not leave their Bluetooth on, all 70% (21/30) indicated phone battery concerns as their reasoning.

A total of 47% (14/30) reported losing the watch, a higher number than what we had recorded, but most of those outliers explained that the watch had fallen off during sport and been found afterwards. A total of 3 of those participants mentioned that they felt that the strap opened too easily. Of the 9 responders who reported forgetting their watch somewhere, 6 (67%) reported that they would often leave it in their sport bag after training.

#### Attitude Toward Use (Open-Ended 1 and 2)

In describing the best thing or things about the watch, 50% (n=15) mentioned sleep tracking, and 57% (n=17) mentioned step and activity tracking. A total of 2 responders reported that the best aspect was achieving their daily step goals, whereas 2 enjoyed having the clock function, and other participants reported the app itself and the style of the device.

Whereas, when describing the worst thing(s) about the watch, 40% (n=12) reported that there was none, 17% (n=5) reported syncing the device, 17% (n=5) reported poor strap security, 10% (n=3) reported discomfort at night, with a further 6 mentioning aspects such as aesthetics, difficulty reading the time, inaccuracy, and forgetting to wear the watch.

#### Behavioral Intention to Use (Open-Ended 3)

In stating what might motivate participants to wear the watch, 37% (n=11) reported tracking their steps and sleep, 13% (n=4) said that nothing would motivate them to wear it, 10% (n=3) reported that an activity goal or competition would be motivating, 2 responders wanted it to be more comfortable, 2 wanted information on calories and energy expenditure, and further responses concerned the need for a better strap for training, a digital clock, a heart rate monitor, more information related to performance, and the watch being of better quality.

#### Actual Use: Follow-Up Questions

Of the 43% (n=13) who reported that they have continued to wear the watch, 9 reported that they find it useful for tracking steps and sleep, 2 reported that they use it primarily for telling the time, and 1 reported that they have become used to using it.

Whereas of the 57% (n=17) who have not continued wearing the watch, 6 reported that they lost the watch, 3 reported that they normally do not wear watches, 3 reported that they wear another watch, 2 reported that it was uncomfortable, 2 reported that they have no use for it, and 1 responder commented that they had forgotten about it.

## Discussion

### Disconnection and Field Test

In this study, the disassociation event had a sizeable impact on how the remainder of the data collection was conducted. Initial deployment of the wearable was designed to act as a field test, whereby we could identify and tackle issues as they arose. This field test consequently developed into the entire data collection phase, molded by the gravity of the initial disassociation and our understanding of how this might impact the participants. Considering the survey results reported only 23% (n=7) having an issue with their watch, we feel that we may have overestimated the potential burden of the disassociation event.

It is not clear whether a field test with a sample of the participants would have prevented the disassociation (which appeared to be a once-off event). However, it is possible that it may have provided insights to modify the study design or preempt other challenges. Certainly, we feel that the issue with device comfort and strap security experienced during sport could have been identified through a field test, which may have enabled us to implement a stronger contingency plan to mitigate wear disruption. However, this prior knowledge may not have affected our ability to regulate device wear during and after training because of the personnel and time it would have required. Under the study conditions, the lead researcher was the sole point of contact for participants while also acting as the team physiotherapist during her regular interaction with participants.

Participant contact from the lead researcher when no data were recorded changed from daily to weekly, corresponding to strategies previously employed [14]. The burden of this monitoring and subsequent contact was evidently time-consuming but also a necessary component when dealing with wearable technology. Weekly checks were less burdensome than daily ones but also allowed for the increased possibility of data loss. In hindsight, as our survey results suggest, the daily reminders were not as burdensome for the participant (19/30 disagree or strongly disagree) as we may have perceived, suggesting this is a more favorable option when feasible.

Regarding our technical challenges with disconnection, API access, and product support, of significance is that Nokia were in the process of evaluating their digital health business at the time, announcing a strategic review and ultimately selling their digital health sector back to Withings from whom they had purchased it in 2016. The availability of product support and customer confidence in the maintenance of data access is of utmost importance when investing financial, time, and human resources in a research project but will not always be predictable for researchers.

### Logistics and Syncing

As the lead researcher worked clinically with this cohort an average of 3 times per week, it was envisaged at the beginning of the study that identifying and rectifying technical or other issues would be relatively easy with this level of access. However, when issues arose, such as a disconnection, a syncing problem, or the loss of a device, participants were generally quite slow to report them or were less likely to make outside contact immediately when an issue was identified. In some cases, participants were not aware of the issue because the Health Mate app had now connected to display their phone’s step count. In other cases, such as a loss, participants were familiar to the lead researcher and may have felt guilty that they had lost the device. In these scenarios, the fact that participants did not sync the watch daily meant that issues were not always readily identifiable on our part.

The survey identified the variety of syncing strategies employed by the participants, with only 23% having a daily syncing routine and no participants reporting that the device synced automatically, as originally purported by Nokia. This resulted in greater burden for the participants while pairing the device, a practice that has been previously shown to negatively influence the sustained use of wearable devices [15]. To avoid scenarios where user maintenance negatively effects data quality, device syncing should be seamless and passive for the participant. A defined and robust syncing strategy is central to success.

When such issues were eventually recognized, simple barriers could make them slow to fix. For example, the assumptions we made that the participants would always carry their phones and that they would be charged and have on-going access to the internet proved to be untrue. In reality, the cohort were busy, used many social media apps, and had to travel long distances for training both of which meant drained batteries were common, whereas internet access was sometimes a problem because of phone data packages. These results challenge our understanding of the preconceived ease of using technologies with young adults, who in fact appear to display their own obstacles in the digital divide [16]. The practical learnings from these issues include the researcher being armed with multiple phone chargers and having a portable Wi-Fi or hotspot setup. In addition, sending extra reminders to participants to bring their device, and if feasible fully charged, is advised when their attendance at a venue is not for the sole purpose of troubleshooting device issues, for instance, they may be attending training (as in this case) or a medical appointment.

Moreover, a number of participants changed their mobile phone over the course of the study—a basic issue that we did not foresee and were generally not preinformed about. If the participant had changed their phone and not synced the device for several days or weeks, those data were lost. This particularly happened around the Christmas period, and these are important challenges to be aware of when using the bring-your-own-device strategy in longitudinal data collection. The alternative being obviously higher cost and also the disruption of employing a device that the participant doesn’t already use daily for other purposes [17].

### Wearing

The results of this study demonstrate that device wear during contact sport was simply not suitable. Although the authors believed the strap of the device to be sturdy and secure, this belief was not shared by participants with regards to contact sport. Results of general comfort were quite favorable but dropped considerably when asked about comfort during sport. These findings are consistent with previously published reasons for taking off smart watches: discomfort during sport and concerns about breakage of the device [18]. Perhaps no watch-like activity tracker is suitable for wear during a physical contact sport. and this is probably reflected in the illegality of same during competitive games. However, contact sports are a common part of life, and when dealing with an active cohort, this is an eventuality that may need to be planned for. In addition, for an active cohort such as this, permanent marking of devices would be necessary, for example, engraving a participant code into the strap.

As mentioned, the disruption of nonwear during training meant that many participants would either forget to take off or put back on the device, and unfortunately, we did not have the resources to regulate this under the study conditions. If this eventuality could have been planned for, we might have taken the initiative of 1 participant who was observed putting the device in his shoe rather than in his sports bag, as a reminder to put it back on his wrist afterwards. A basic gesture but an effective one nonetheless.

### Perceptions and Attitudes

Although there were many challenges involved in this data collection, if we were to evaluate adherence to the device based on the wear characteristics of the remaining 18 participants, we could conclude that this study was successful. However, the issues experienced meant that our cohort size was depleted, in addition to the original 14-week split observation being reduced to 1 12-week observation. When asked if they needed more information about the watch or the app, only 1 responder reported that they did, which appears to reflect that the cohort felt well-informed and confident in their use of the device and negates many of the current adoption challenges on the other side of the digital divide [19]. The open-ended responses regarding the best thing about the watch and what might motivate them to continue wearing the watch had a common theme relating to the participants desire for more information related to their health and performance. These answers appear to reflect the interests of a group of sportspeople who want to better their sporting performance and understand how their activity and sleep can influence this, which follows previous research underpinning motivation and feedback as leading facets of engagement [20,21]. However, the participant characteristics should be considered in these interpretations: as an already active group of males, they may be more likely to use a wearable activity tracker than other cohorts [22]. There was poor agreement as to whether the team had a competitive attitude to step counting or not, which probably reflects that only some individuals treated it as a competition. This is important to note with regards to its effect, or lack thereof, on user motivation and compliance.

### Limitations

A limitation of the presented study has been mentioned throughout; the fact that we did not set out to investigate and report on a specific research question; it is rather, an unstructured, retrospective evaluation of another study. Therefore, this report does not follow the standard layout and presentation of results that one might expect.

Although this study presents common issues that can be experienced in a wearable deployment, it is important to consider that this case study represents a single regional cohort using a specific activity tracker and mobile app. The cohort were a small, purposeful sample chosen for the requirements of the original study and cannot be assumed to represent a general population.

The authors acknowledge the limitations of the survey, although anonymous, the participants’ familiarity with the lead researcher may have affected their honesty in reporting on their experience.

### Conclusions

Our findings contrast many of the published barriers to wearable use such as discomfort [23] and technology familiarity [24] and support many referenced facilitators such as user acceptance and perceived usefulness [23]. Nevertheless, our data collection was highly impacted by challenges, namely logistics, technical issues, and loss of the device. These factors are not always predictable and will need to be strongly considered to ensure satisfactory data quality is achieved. It is reasonable to suggest that some of the issues we encountered might have been mitigated with careful planning and pilot studies. Nonetheless, the frequency and range of issues encountered did not reflect the level of preparatory work that was done. Previously published potential solutions to the user adherence challenge lists many of the strategies employed in this study, such as providing clear instructions, reminders, and checking in with participants regularly to identify issues [25]. On the basis of our experience, we suggest readers to consider their research context in the selection of a consumer wearable [26], including the availability of an extensive support team to undertake these requirements. Issues will arise despite extensive preparation and exacting due diligence, and they will need to be identified and rectified promptly to maintain data quality. Although we do not expect exact replication of these challenges in other contexts, our experience can be a useful example for learnings in a wider readership for wearables research.

### Learning Outcomes and Recommendations

#### The Field Test

Although our field testing did not work out as planned, we would recommend field testing your device with a subset of the cohort to identify practical and technical issues that may arise. This will enable the refinement of implementation and adoption strategies that align with the needs of your cohort.

#### The Syncing Strategy

Passive (automatic) and seamless device syncing is optimal for user compliance and maintained use as it facilitates immediate identification of issues such as nonwear or disconnection. If automated syncing and upload is not possible, the strategy should include clear instructions for users to perform manual sync on a daily basis.

#### Bring-Your-Own-Device

When using a participant-owned mobile phone, the deployment team should take time to adjust the phone settings to ensure that no partner apps can share and extract data, such as access to the phone’s step counter. This unsolicited data sharing could conceal technical issues and may be indiscernible from activity tracker data when the raw dataset is downloaded. It is essential to plan for and be informed about personal mobile phone changes from participants.

#### Support Team and Logistics

With such a sizeable time-burden involved in monitoring daily syncing, it is imperative to invest in the appropriate resources to monitor this wear and resolve issues. When dealing with a working population, it may be difficult to contact participants during the day, and home visits may also be unavailable to you. Although we had regular access to the participants, resolving issues did not work as smoothly as planned, partly because the access was not for the sole purpose of partaking in the study.

## Multimedia Appendix 1

Participant survey.

## Multimedia Appendix 2

Survey results.

## Abbreviations

API: application programming interface
