# Supplementary material for: Consumer Wearable Deployments in Actigraphy Research: Evaluation of an Observational Study
Source: JMIR Mhealth Uhealth. 2019 Jun 24;7(6):e12190. doi: 10.2196/12190 (PMC6613323; doi:10.2196/12190)
Supplement: Supplementary file 1 [file mhealth_v7i6e12190_app1.pdf]

Multimedia Appendix 1: Participant Survey

1. Do you normally wear a watch every day?

☐ Yes

☐ No

2. Do you normally leave your phone Bluetooth on?

☐ Yes

☐ No

3. Please outline briefly why you do or don't leave your Bluetooth on.

4. The watch effected me in work/college.

☐ Strongly disagree

☐ Disagree

☐ Neutral

☐ Agree

☐ Strongly agree

5. If you agree or strongly agree, please outline how the watch affected you:

6. The watch was comfortable during the DAY.

☐ Strongly disagree

☐ Disagree

☐ Neutral

☐ Agree

☐ Strongly agree

7. The watch was comfortable at NIGHT.

☐ Strongly disagree

☐ Disagree

☐ Neutral

☐ Agree

☐ Strongly agree

8. The watch was comfortable during TRAINING.

☐ Strongly disagree

☐ Disagree

☐ Neutral

☐ Agree

☐ Strongly agree

9. I enjoyed tracking my STEPS.

☐ Strongly disagree

☐ Disagree

☐ Neutral

☐ Agree

☐ Strongly agree

10. I enjoyed tracking my SLEEP.

☐ Strongly disagree

☐ Disagree

☐ Neutral

☐ Agree

☐ Strongly agree

11. My STEP count was accurate.

☐ Strongly disagree

☐ Disagree

☐ Neutral

☐ Agree

☐ Strongly agree

12. My SLEEP duration was accurate.

☐ Strongly disagree

☐ Disagree

☐ Neutral

☐ Agree

☐ Strongly agree

13. I found it useful tracking my steps/sleep.

☐ Strongly disagree

☐ Disagree

☐ Neutral

☐ Agree

☐ Strongly agree

14. If you agree or strongly agree, please outline briefly how you found it useful:

15. There was a competitive aspect on the team for the highest step count.

☐ Strongly disagree

☐ Disagree

☐ Neutral

☐ Agree

☐ Strongly agree

16. Did you ever lose your watch?

☐ Yes

☐ No

17. If you lost the watch, please outline how:

18. Did you ever forget your watch somewhere?

☐ Yes

☐ No

19. If yes, please outline why or how you forgot the watch:

20. Did you ever confuse your watch with someone else's?

☐ Yes

☐ No

21. Did you have any issues with your watch?

☐ Yes

☐ No

22. If yes, please outline what the issue was:

23. How often did you sync the watch?

☐ Daily

☐ Once a week

☐ When I remembered

☐ When I was reminded

☐ The watch synced automatically

24. I found the reminders to sync the watch annoying.

☐ Strongly disagree

☐ Disagree

☐ Neutral

☐ Agree

☐ Strongly agree

25. Did you have any issues syncing the watch?

☐ Yes

☐ No

26. If yes, what was the issue?

27. I needed more information about the watch/app.

☐ Strongly disagree

☐ Disagree

☐ Neutral

☐ Agree

☐ Strongly agree

28. If you agree or strongly agree, what would you like to have known?

29. Please outline the best thing about using the watch.

30. Please outline the worst thing about using the watch.

31. Have you continued wearing the watch?

☐ Yes

☐ No

32. Why/why not?

33. What might motivate you to continue wearing the watch?

34. If you have any other comments please leave them here. Thank you.
