# Supplementary material for: Consumer Wearable Deployments in Actigraphy Research: Evaluation of an Observational Study
Source: JMIR Mhealth Uhealth. 2019 Jun 24;7(6):e12190. doi: 10.2196/12190 (PMC6613323; doi:10.2196/12190)
Supplement: Supplementary file 2 [file mhealth_v7i6e12190_app2.pdf]

Multimedia Appendix 2: Survey Results

**Practical considerations**		**Yes, n (%)**	**No, n (%)**
	Do you normally wear a watch every day?	16 (53)	14 (47)
	Do you normally leave your phone Bluetooth on?	9 (30)	21 (70)
	Did you ever lose your watch?	14 (47)	16 (53)
	Did you ever forget your watch somewhere?	9 (30)	21 (70)
	Did you ever confuse your watch with someone else's?	3 (10)	27 (90)
	Did you have any issues with your watch?	7 (23)	23 (77)
	Did you have any issues syncing your watch?	2 (7)	28 (93)
**Actual use**	Have you continued wearing the watch?	13 (43)	17 (56)

		**Daily, n (%)**	**Once a week, n (%)**	**When reminded, n (%)**	**When I remembered, n (%)**	**Auto-sync, n (%)**
**Actual use**	How often did you sync the watch?	7 (23)	5 (17)	12 (40)	6 (20)	0

		**Strongly agree, n (%)**	**Agree, n (%)**	**Neutral, n (%)**	**Disagree, n (%)**	**Strongly disagree, n (%)**
**Perceived ease of use**	The watch affected me in work/college.	1 (3)	2 (7)	3 (10)	10 (33)	14 (47)
	The watch was comfortable during the DAY.	10 (33)	17 (57)	2 (7)	0	1 (3)
	The watch was comfortable at NIGHT.	9 (30)	10 (33)	7 (23)	3 (10)	1 (3)
	The watch was comfortable during TRAINING.	5 (17)	10 (33)	6 (20)	9 (30)	0
	I found the syncing reminders annoying.	0	1 (3)	10 (33)	15 (50)	4 (13)
**Perceived usefulness**	I enjoyed tracking my STEPS.	8 (27)	19 (63)	3 (10)	0	0
	I enjoyed tracking my SLEEP.	10 (33)	18 (60)	2 (7)	0	0
	My STEP count was accurate.	6 (20)	20 (67)	3 (10)	1 (3)	0
	My SLEEP duration was accurate.	7 (23)	15 (50)	6 (20)	2 (7)	0
	I found it useful tracking my steps/sleep.	8 (27)	15 (50)	6 (20)	1 (3)	0
**Attitude toward use**	There was a competitive aspect for the highest step count.	0	11 (37)	12 (40)	7 (23)	0
	I needed more information about the watch/app.	0	1 (3)	10 (33)	14 (47)	5 (17)
